# Molecular detection, infection rate and vectors of *Theileria lestoquardi* in goats from West Azerbaijan province, Iran 

**Published:** 2017-06-15

**Authors:** Seyyed Mostafa Mohammadi, Bijan Esmaeilnejad, Ghader Jalilzadeh-Amin

**Affiliations:** 1 *Graduate Student, Faculty of Veterinary Medicine, Urmia University, Urmia, Iran;*; 2 *Department of Pathobiology, Faculty of Veterinary Medicine, Urmia University, Urmia, Iran; *; 3 *Department of Internal Medicine and Clinical Pathology, Faculty of Veterinary Medicine, Urmia University, Urmia, Iran.*

**Keywords:** Goat, Iran, Ixodid ticks, Semi-nested PCR, *Theileria lestoquardi*

## Abstract

This study was aimed to determine the infection rate and vectors of *Theileria lestoquardi *in goats from West Azerbaijan province, Iran. A total of 400 blood samples were collected from 40 randomly selected flocks in the study area from June to September, 2014. Out of 400 blood samples examined using microscopic examination, a number of 14 goats (3.50%) were positive for *Theileria* spp*.*, whereas 25 goats (6.25%) yielded a specific *T. lestoquardi *SSU-rRNA fragment (235 bp). The prevalence of theileriosis in goats estimated by semi-nested PCR was significantly higher than the prevalence estimated by microscopic examination of the blood smears. The prevalence of *Theileria* infection in different age and sex groups of goats was not significantly different. The highest and lowest prevalence of *Theileria* infection was in July (12.00%) and September (2.00%), respectively. A number of 315 adult Ixodid ticks were also collected from naturally infested goats and they were characterized. Out of 315 examined ticks, a number of 37 ticks including *Hyalomma marginatum *(65.20%), *Rhipicephalus turanicus *(44.00%), and *Dermacentor marginatus *(68.70%) were infected by *T. lestoquardi*. Based on the obtained results, it was concluded that the semi-nested PCR assay based on SSU-rRNA gene is a valuable method for epidemiological investigation of caprine theileriosis. The results showed that *H. marginatum*, *R. turanicus *and *D. marginatus *can be considered as risk factor in the epidemiology of *T. lestoquardi*.

## Introduction

Tick-borne diseases are caused by infection with a variety of pathogens, including rickettsia and other types of bacteria, viruses, and protozoa.^1^ Theileriosis is a hemoparasitic tick-borne disease of domestic and wild animals caused by different species of the genus *Theileria*.^2^

*Theileria lestoquardi*, *T. uilenbergi *and *T. luwenshuni* cause malignant theileriosis in sheep and in some cases in goats.^3^ The other species, *T. ovis* and *T. separata*, cause subclinical infection in small ruminants.^4^ Two species, *T. lestoquardi* and *T. ovis* are suspected to cause ovine theileriosis in Iran.^2^ *Theileria lestoquardi*, which is a causal agent of malignant sheep and goat theileriosis, was frequently reported from different parts of Iran.^5^^,^^6^ Symptoms of acute disease include fever, anemia and jaundice and in some cases mortality may occur.^7^ Ixodidea ticks including *Rhipicephalus* spp. and *Hyalomma *spp. have been implicated in the transmission of small ruminants' theileriosis.^8^ The economic losses in sheep and goats production due to theileriosis are significant in tropical and subtropical areas.^9^^,^^10^ Diagnosis of theileriosis can be achieved by microscopic examination of Giemsa-stained blood smears and clinical signs in acute phase of the disease, but after acute infections, recovered animals frequently sustain subclinical infections, which are difficult to detection microscopically.^11^ Subclinical infections can be the sources of transmission of *Theileria* spp. to the vectors and subsequently to the susceptible hosts.^12^ Although the examination of blood smears is used routinely, some problems may appear to give accurate diagnosis and identify *Theileria* spp.^13^ The use of alternative techniques, such as polymerase chain reaction (PCR), has become necessary to detect and identify *Theileria* infections effectively and has been reported in numerous recent studies.^12^^,^^14^^,^^15^ Molecular techniques are more sensitive and specific than other traditional diagnostic methods. Recently, DNA amplification methods have been developed and used for the detection of *Theileria* spp.^13^^,^^15^ Molecular identification of *T. ovis* infection in sheep and parasitized ticks was previously performed in northern Iran,^16^ as well as in adjacent countries e.g. Turkey^17^^,^^18^ and Iraq.^19^ However, presence of *T. lestoquardi *in the collected ticks from goat and blood samples has not been simultaneously detected by semi-nested PCR. To our knowledge, this is the first study designed to investigate the malignant theilariosis in goats and identify its tick vectors in West Azerbaijan, Iran. Additionally, comparison was made between the infection rate of *T*. *lestoquardi* determined by semi-nested PCR and that of determined by thin blood smears. 

## Materials and Methods

**Study area. **The study was carried out in West Azerbaijan province, which was located in an important livestock production region in the northwest of Iran. This region was divided into three different geographical areas, namely north, center and south. Ecologically, this area is classified as a semi-arid zone. Livestock farming is an important economic sector in this province.

**Collection of blood and tick samples.** Considering the expected prevalence of 50.00% and 5.00% absolute precision with 95.0% confidence level, a total of 400 blood samples were collected from Marghoz breed goats that belonged to 40 randomly selected flocks during favorable seasons, from early June to September of 2014. All of the flocks were maintained outside and only brought into the paddock during the night. The animals freely grazed on natural pasture and were not received any supplement. At least eight animals were randomly chosen from each flock. Flocks were divided into two size categories: Flocks with 20-100 animals and more than 101 animals. Animals were also grouped into two age categories: less than one year-old (< 1 year) and above one-year old (≥ 1 year). During sampling, the whole body of each goat was examined for the presence of ticks by palpation, mainly on their ears, along their nape of neck, perineum, and udder/orchid, between thighs, shoulder region and tail base. The ticks were manually removed and transferred to the parasitology laboratory in tubes containing 70% ethanol. A total of 315 adult ticks were collected from examined animals.

**Examination of blood smears and ticks. **The thin blood smears which prepared from ear capillaries were fixed in methanol for 5 min and stained with 10.00% Giemsa solution in phosphate buffer solution (PBS), pH 7.2, for 20 min. The blood smears were examined under an oil-immersion objective of a magnification of 1000 × for the presence of intracellular forms of the parasite with morphology compatible with *Theileria *spp.^13^ The percentage of infected red cells was calculated. For the estimating parasitemia, 100 microscopic fields containing approximate 1000 red blood cells per field were reviewed and the number of the parasites per 100,000 red blood cells was enumerated.^8^ The smears were assigned as negative for all hemoparasites if no piroplasms were observed in a 100-oil-immersion field. The collected ticks from animals were counted. Ticks' species were identified using the standard taxonomic keys.^20^^,^^21^

**DNA extraction from blood samples and ticks. **DNA extraction was performed according to the procedure described by Clausen *et al*. with some modifications.^22^ Briefly, 125 µL of each blood sample was added to 250 µL of lysis buffer (0.32 M sucrose, 0.01 M Tris, 0.005 M MgCl_2_, 1.0 % Triton X-100, pH 7.50) and the mixture was centrifuge at 11600 *g* for 1 min. The pellet was washed three times by centrifugation with 250 µL lysis buffer. The supernatant was discarding and the final pellet was resuspended in 100 µL of PCR buffer [50 mM KCl, 10 mM Tris-HCl (pH 8), 1.0%, Triton x-100, pH 8.30] containing 50 µg of proteinase K per mL and then incubated at 65 ˚C for 1 hr. Finally, the sample was boiled at 95˚C for 10 min.

In order to detect *Theileria* DNA in ticks, the stored ticks in 70% ethanol were subjected for DNA extraction. Ticks were removed from 70% ethanol and air-dried on a filter paper. Salivary glands of each tick were dissected out and transferred into sterile PBS (pH 7.4). DNA was extracted from salivary glands of each tick according to the procedure described by Das and Ray.^23^ Briefly, the removed salivary gland was homogenized in 400 µL of homogenizing buffer (0.4 M NaCl, 10 mMTris-HCl, 2 mM EDTA, pH 8) and then mixed with sodium dodecyl sulfate (Merck, Darmstadt, Germany) (2% final concentration) and proteinase K (400 μg mL^-1 ^final concentration). The resultant mixture was incubated at 56˚C for 2 hr, after which 300 µL of 6 M NaCl was added to the sample. The sample was vortexed for 30 sec, and centrifuged at 12000 *g*. The supernatant was transferred to a new tube and an equal volume of isopropanol was added to each sample, mixed well, and samples were incubated at –20˚C for 1 hr. Samples were then centrifuged for 20 min at 10000 *g*. The pellet was washed with 70% ethanol, dried and finally resuspended in 50 to 100 µL sterile dH_2_O.

**PCR and semi-nested PCR amplification. **In order to detect *T. lestoquardi *in blood samples and ticks, a pair of primers, Theil-F 5'-CACAGGGAGGTAGTGACAAG-3' and Bab-R 5'- AAGAATTTCACCTCTGACAG-3' were used to amplify a 426-430 bp fragment of the SSU-rRNA gene of *Theileria *spp. The primer's specificity and sensitivity was assessed by by Shayan and Rahbari.^11^ An amount of 10 ng of first PCR product was then subjected to semi-nested PCR using the additional primer, 5'-ATTGCTTGTGTCCCT CCG-3'. The semi-nested PCR was carried out in 50 µL total reaction volume containing 5 µL of 10 x PCR buffer, 2 mM MgCl_2_, 250 µM of each of the four deoxynucleotide triphosphate, 1.25 U Taq DNA polymerase (Fermentas, Hamburg, Germany), 50 pmol of each primer, and 10 ng of amplified PCR extracted DNA. Amplification of parasite DNA was performed in a Corbett thermocycler (Model CP2-003; Corbett Research, CP2-003, Sydney, Australia). Cycling condition included an initial denaturation at 95˚C for 5 min, followed by 35 cycles at 94˚C for 45 sec, 55˚C for 90 sec and 72˚C for 45 sec. Finally, semi-nested PCR was completed with the additional extension step for 5 min in 72˚C. The PCR products were separated by electrophoresis on 2% agarose gel in Tris-Borate-EDTA (Merck) buffer and visualized using ethidium bromide (1 μg mL^-1^) and UV transilluminator (model BTS-20M; Uvitec, Cambridge, UK). The positive control for *T. lestoquardi* was kindly provided by Dr. Abbas Imani from Faculty of Veterinary Medicine, University of Tabriz, Tabriz, Iran. 

**Statistical analysis. **Chi-square test was used for analyzing of infection rates. The exact Fisher test was also used to express association between the presence of *Theileria* and the various parameters i.e. flock size, gender and age of animal, tick infestation of goats, presence and tick infestation of flock. A *p*-value less than 0.05 was considered significant.

## Results

**Fig. 1:**
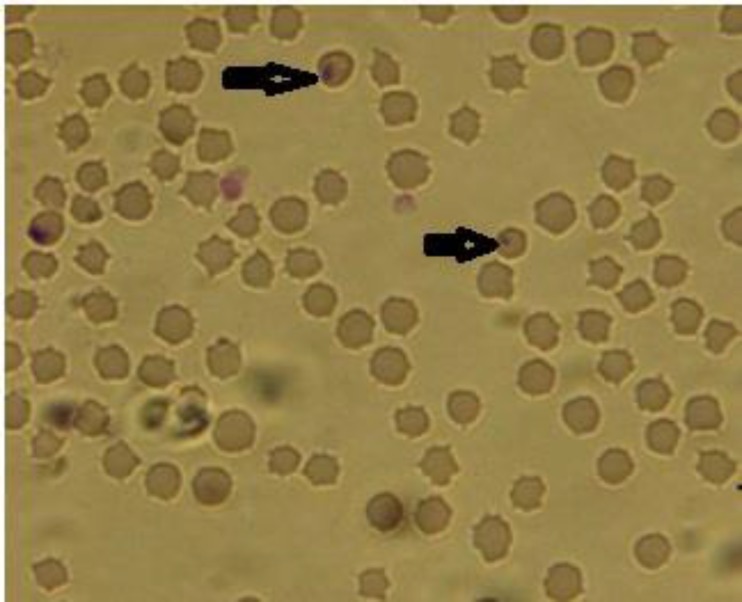
*Theileria* spp. (arrows) inside an infected goat’s erythrocytes in a blood smear (Giemsa, 100

**Microscopic examination. **Microscopic examination of thin blood smears showed parasitemia in infected animals ranging from 0.01 to 0.20% piroplasms, detected inside the red blood cells, showing round, oval and single ring shapes (Fig. 1). All of these forms were classified as *Theileria *spp. Microscopically, a number of 14 (3.50%) blood samples out of 400 examined blood smears were positive for piroplasms.

**Fig. 2:**
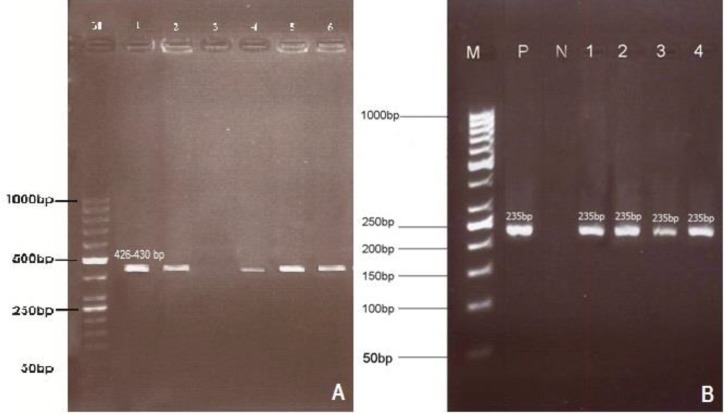
First round PCR (A) and semi-nested PCR (B) amplified products using *Theileria lestoquardi* specific primers. Lane M- 50bp DNA ladder (Fermentas, Germany), A) Lane 1: Positive control, Lane 2: Infected goat blood, Lane 3: Negative control, Lane 4: Infected *H.* *marginatum*, Lane 5: Infected *R. turanicus*, Lane 6: Infected *D. marginatus*. B) Lane P: Positive control, Lane N: Negative control, Lanes 1, 2: Infected goats blood, Lanes 3, 4: Infected ticks

**Detection of** ***T. lestoquardi*** **using PCR and semi-nested PCR. **All obtained blood samples were examined by PCR procedure amplifying a 426-430 bp fragment of SSU-rRNA gene of *Theileria *spp., and the semi-nested PCR was employed for determination of *T. lestoquardi *infection (235 bp). The results showed that 25 (6.25%) goats were infected (Fig. 2). Among 40 examined flocks, *T. lestoquardi* infection was detected in twelve (30.0%) flocks. All positive samples of goats by microscopic examination were also positive in semi-nested PCR. The prevalence of *T. lestoquardi* in goats in relation to the parameters describing the characteristics of the animals and the flocks is showed in Table 1.

**Tick infestation of examined animals. **During the sampling period (June-September 2014), a total of 315 adult Ixodid ticks were collected from sampled goats. The main attachment site of ticks was the perineum region. Following ticks were isolated, *Hyalomma anatolicum *(58.70%)*, H. marginatum *(7.30%)*, Rhipicephalus bursa *(14.60%)*, R. sanguineus *(4.50%)*, R. turanicus *(8.00%)*, Dermacentor marginatus *(5.10%) and *Haemaphysalis punctata *(1.90%), (Table 2).

**Table 1:** Association between the presence (PCR-positive and negative blood samples) of *T. lestoquardi* infection in goats and the studied parameters describing animal and flock characteristics. The data within the parentheses are presented as percentage

**Parameters**	**Total **	**Flock size**		**Gender **		**Age **		**No. of tick burden **	
		**20-100 **	**> 100 **	**Male**	**Female**	**< 1 year**	**≥ 1 year**	**No tick**	**≥ one Tick**
**Number**	400	320	80	100	300	125	275	315	85
**Negative**	375 (93.75)	307 (96.00)	681 (85.00)	94 (94.00)	281 (93.70)	114 (91.20)	261 (94.90)	305 (96.80)	70 (82.00)
**Positive**	25 (6.25)	13 (4.00)	12 (5.00)	6 (6.00)	19 (6.30)	11 (8.80)	14 (5.10)	10 (3.2.00)	15 (17.00)
**P(F)**		0.09 (NS)		0.41 (NS)		1.00 (NS)		0.003	

P(F): Fisher's exact test; NS: Not significant.

**Table 2:** Frequency of tick species on the infected goats and percentage of ticks infected with *T. lestoquardi *using semi-nested PCR. The data within the parentheses are presented as percentage

**Tick Species**	**Tick number **	**Male**	**Female**	**Total infected ticks **	**Infected male**	**Infected female **
***Hyalomma anatolicum***	185 (58.70)	106	79	-	-	-
***H. marginatum***	23 (7.30)	14	9	15 (65.20)	5 (21.70)	10 (43.50)
***Rhipicephalus bursa***	46 (14.60)	37	9	-	-	-
***R. sanguineus***	14 (4.50)	9	5	-	-	-
***R. turanicnus***	25 (8.00)	16	9	11 (44.00)	6 (24.00)	5 (20.00)
***Dermacentor marginatus***	16 (1.90)	12	4	11 (68.50)	8 (50.00)	3 (18.75)
***Haemaphysalis punctata***	6 (1.90)	3	3	-	-	-
**Total**	315	197	118	37 (11.70)	19 (6.30)	18 (5.41)

**Detection of** ***T. lestoquardi*** **in ticks by semi-nested PCR. **Amplified PCR product of *Theileria *spp. from salivary glands of ticks is 426-430 bp. Subsequently, by semi-nested PCR an expected 235 bp fragment of *T. lestoquardi *was amplified from 37 out of 315 (11.70%). *Theileria lestoquardi *was detected in salivary glands of *H. marginatum *(65.20%), *R. turanicus *(44.00%) and *D. marginatus *(68.70%). The difference of infection rate for male and female ticks was not statistically different (*p *> 0.05), (Table 2). Frequency of *T. lestoquardi* infection was significantly higher in flocks with tick burden than flocks without tick burden (*p* < 0.05), (Table 1). 

The monthly related prevalence of infection in goats and infected ticks was the highest in July, while a decrease was observed in September (*p *< 0.05). The July peak in prevalence of infection was correlated with tick numbers (Table 3).

**Table 3:** Prevalence of *Theileria* infection by month in goats of west-Azerbaijan province, Iran

**Month**	**No. of goatssampled**	**Frequency of positive samples for ** ***Theileria*** ** spp. (%)**
**June**	95	4 (4.20)
**July**	100	12 (12.00)
**August**	125	6 (4.80)
**September**	80	2 (2.50)
**Total**	400	25 (6.25)

## Discussion

Small ruminant farming is one of the main animal husbandry activities in the north west of Iran. The presence of potential vectors, ticks, and susceptible hosts for caprine malignant theileriosis in all parts of Iran pose a real threat to food animal industry. The occurrence of small ruminant’ theileriosis especially ovine theileriosis had been previously reported in Iran, but prevalence of *Theileria *spp. in goats has not yet been determined.^6^

Microscopic examination, mainly Giemsa-stained blood smears is commonly used as a confirmatory diagnosis of vertebrate hosts suffering from piroplasm infections. However, the method requires expertise because these parasites have similar morphological features and therefore, may confuse the examiner when mixed infections occur. Serological tests were also used, but there are some difficulties with specificity and sensitivity.^24^ An exact differentiation between these parasites is crucial to understanding their epidemiology. The detection of *Theileria* infection in carrier animals by DNA amplification has been a powerful tool for epidemiological investigation, since these animals represent an important source of alimentary infection of ixodidae ticks.^17^^,^^25^

Although PCR assays for the detection of small ruminant theileriosis have been published previously, the present study was the first molecular diagnostic technique that was employed to determine infection rate of *T. lestoquardi *in goats and vector ticks in Iran. Molecular techniques such as PCR have higher efficiency than micro-scopic examination and serological assays for detection of *T. lestoquardi.*^6^^,^^15^^,^^25^^-^^27^

In the microscopic examination, it was found that parasitemia ranged from 0.01 to 0.20%. In similar study, Razmi *et* *al*. has observed that sheep infected with *Theileria *spp. commonly had low parasitemia.^6^ The highest prevalence of *Theileria* infection was observed in summer in which the population of tick is high. Previous studies have shown that the rise of infection with *Theileria *spp. was related to the seasonal activity of vector ticks.^6^^,^^8^ No significant association between age and sex of the animals and *Thieleria *spp. infection has been reported in naturally infected sheep and goats with *Thieleria *spp.^6^^,^^28^

In the present study, *T. lestoquardi *infection was only detected in eight animals (11.70%). The low prevalence of *T. lestoquardi *found in this study is same the results obtained earlier.^17^^,^^29^ In contrast with our results, higher infection rate with three species of *Theileria*, including *T. lestoquardi*, was reported in sheep in the same area by Zaeemi *et al*.^29^ This difference might be due to higher infestation of sheep with ticks in their study; furthermore, higher numbers of ticks harbor *Theileria* spp. It could be speculated that there might be a difference between the susceptibility of sheep and goat to infection with *Theileria* in Iran. However, this point needs more investigation.

A problem discussed in protozoan infections is the determination and characterization of the vetors.The examination of ticks’ salivary glands stained with Methyl-green-pyronin (MGP) or Feulgen methods may reveal the presence of sporozoites, but this method lacks high sensitivity and specificity and/or is time-consuming and the difficulty in differentiating the species involved, thus, the transfer vector remains unanswered.^6^^,^^8^^,^^15^ Therefore, the specific and sensitive alternative techniques such as species-specific PCR methods have been developed and used for the detection of *Theileria* spp.^25^ In our study, *H. anatolicum, H. marginatum, R. bursa, R. sanguineus, R. turanicus, D. marginatus *and *H. punctata *were identified from goats, and all of them were examined by semi-nested PCR. Among these ticks, *T. lestoquardi* was detected in *H. marginatum *(65.20%), *R. turanicus *(44.00%) and *D. marginatus *(68.70%) from naturally infested goats. Similar findings have been reported by previous PCR-based studies.^5^^,^^8^^,^^30^^-^^32^ However, in contrast with our results, Razmi *et al*. reported that *H. anatolicum *infestation was more frequent than *H. marginatum *in Khorasan province, Iran.^8^ This may be due to geographical disparity between two regions, different techniques that were used for staining and long time span between both studies may have been resulted in a better adaptation of *H. marginatum *to local climate condition. The most of the tick species were found on the perineal region of examined goats that is similar with the results obtained by Mazlum and Razmi *et al*.^8^^,^^30^

The finding that the prevalence of small ruminant theileriosis was higher in herds with tick burden indicates a positive correlation between the prevalence of the disease and the presence of vector ticks. It was in accordance with the findings of Zaeemi *et al.*^29^ Our results suggest that, the prevalence of Theileria infection was not significantly different between female and male ticks. This result was in agreement with a report by Yin and Luo.^33^

In conclusion, the epidemiology of caprine theileriosis due to *T. lestoquardi* is closely related to the bioecology of ticks and their seasonal activity. The semi-nested PCR assay based on SSU-rRNA gene is valuable method for epidemiological investigation of caprine theileriosis in the northwest of Iran. The results showed that *H. marginatum*, *R. turanicus *and *D. marginatus *could be considered as a risk factor in the epidemiology of *T. lestoquardi*.

## References

[1] Mehlhorn H, Schein E (1984). The piroplasms: Life cycle and sexual stages. Adv Parasitol.

[2] Hashemi-Fesharki R (1997). Tick-borne parasites of sheep and goats and their related vectors in Iran. Parassitologia.

[3] El Imam A H, Taha Kh M (2015). Malignant Ovine theileriosis (Theileria lestoquardi): A review. Jordan J Biol Sci.

[4] Yin H, Schnittger L, Luo JX (2007). Ovine theileriosis in China: A new look at an old story. Parasitol Res.

[5] Hooshmand-Rad P, Hawa NY (1973). Malignant theileriosis of sheep and goats. Trop Anim Health Prod.

[6] Razmi GR, Eshrati H, Rashtibaf M (2006). Prevalence of Theileria spp infection in sheep in South Khorasan province, Iran. Vet Parasitol.

[7] Uilenberg G (1997). General review of tick-borne diseases of sheep and goats worldwide. Parassitologia.

[8] Razmi GR, Hosseini M, Aslani MR (2003). Identification of tick vectors of ovine theileriosis in an endemic region of Iran. Vet Parasitol.

[9] Barnett SF (1974). Economical aspects of tick-borne disease control in Britain. Bull Off int Epiz.

[10] Brown CGD, Ilhan T, Kirvar M (1998). Theileria lesto-quardi and T annulata in cattle, sheep, and goats In vitro and in vivo studies. Ann N Y Acad Sci.

[11] Shayan P, Rahbari S (2005). Simultaneous differentiation between Theileria spp and Babesia spp on stained blood smear using PCR. Parasitol Res.

[12] Altay K, Dumanlia N, Holmanb PJ (2005). Detection of Theileria ovis in naturally infected sheep by nested PCR. Vet Parasitol.

[13] Aktas M, Altay K, Dumanli N (2005). Survey of Theileria parasites of sheep in eastern Turkey using polymerase chain reaction. Small Rumin Res.

[14] Altay K, Aktas M, Dumanli N (2007). Theileria infections in small ruminants in the east and southeast Anatolia. Türkiye Parazitol Derg.

[15] Heidarpour Bami M, Haddadzadeh HR, Kazemi B (2009). Molecular identification of ovine Theileria species by a new PCR-RFLP method. Vet Parasitol.

[16] Telmadarraiy Z, Oshaghi MA, Hosseini Vasoukolaei N (2010). First molecular detection of Theileria ovis in Rhipicephalus sanguineus tick in Iran. Asian Pac J Trop.

[17] Aydin MF, Aktas M, Dumanli N (2013). Molecular identification of Theileria and Babesia in sheep and goats in the Black Sea region in Turkey. Parasitol Res.

[18] Inci A, İca A, Yildirim A (2010). Identification of Babesia and Theileria species in small ruminants in Central Anatolia (Turkey) via reverse line blotting. Turk J Vet Anim Sci.

[19] Alfetly DRH (2012). Detection of Theileria sp In blood samples and estimation of haematological and biochemical changes in sheep in Al-Diwaniya province. Kufa J Vet Med Sci.

[20] Walker AR, Bouattour A, Camicas JL (2003). Ticks of domestic animals in Africa: A guide to identification of species. Edinburgh, Scotland: Bioscience Reports.

[21] Estrada-Peña A, Bouattour A, Camicas JL (2004). Ticks of domestic animals in Mediterranean region, a guide to identification of species.

[22] Clausen PH, Wiemann A, Patzelt R (1999). Use of a PCR assay for the specific and sensitive detection of Trypanosoma spp in naturally infected dairy cattle in peri-urban kampala Uganda. AnnN Y Acad Sci.

[23] Das G, Ray D (2003). PCR-based detection of Theileria annulata infection in ticks collected from cattle of West Bangal, India. J Vet Parasitol.

[24] Passos LM, Bell-Sakyi L, Brown CG (1998). Immunochemical characterization of in vitro culture-derived antigens of Babesia bovis and Babesia bigemina. Vet Parasitol.

[25] Spitalska E, Namavari MM, Shad-del HF (2005). Molecular surveillance of tick-borne diseases in Iranian small ruminants. Small Rumin Res.

[26] Kirvar E, Ilhan T, Katzer F (1998). Detection of Theileria lestoquardi (hirci) in ticks, sheep, goats using polymerase chain reaction. Ann NY Acad Sci.

[27] Schnittger L, Yin H, Gubbels M (2004). Simultaneous detection and differentiation of Theileria and Babesia parasites infecting small ruminants by reverse line blotting. Parasitol Res.

[28] Altay K, Dumanli N, Aktas M (2007). Molecular identification, genetic diversity and distribution of Theileria and Babesia species infecting small ruminants. Vet Parasitol.

[29] Zaeemi M, Haddadzadeh H, Khazraiinia P (2011). Identification of different Theileria species (Theileria lestoquardi, Theileria ovis, and Theileria annulata) in naturally infected sheep using nested PCR-RFLP. Parasitol Res.

[30] Mazlum Z (1972). Tick species of Iran, its distribution, host and seasonal activity [Persian]. J Vet Res.

[31] Abdigoudarzi M (2013). Detection of naturally infected vector ticks (Acari: Ixodidae) by different species of Babesia and Theileria agents from three different enzootic parts of Iran. J Arthropod Borne Dis.

[32] Khodaverdi Azghandi M, Razmi GR (2015). Identification of Babesia and Theileria species in goats and ticks with smear observation and molecular examination in Mashhad, Khorasan Razavi province, Iran. J Vet Res.

[33] Yin H, Luo J (2007). Ticks of small ruminants in China. Parasitol Res.

